# Recurrent lung adenocarcinoma benefits from microwave ablation following multidisciplinary treatments: A case with long-term survival

**DOI:** 10.3389/fsurg.2022.1038219

**Published:** 2023-01-06

**Authors:** Guanyu Jiang, Chenghu Song, Yongrui Xu, Shengfei Wang, Huixing Li, Rongguo Lu, Xiaokun Wang, Ruo Chen, Wenjun Mao, Mingfeng Zheng

**Affiliations:** Department of Thoracic Surgery, The Affiliated Wuxi People's Hospital of Nanjing Medical University, Wuxi, China

**Keywords:** lung cancer, multidisciplinary treatment, target therapy, microwave ablation, immune reaction

## Abstract

Lung cancer has become the leading cause of cancer death all over the world. Nowadays, there is a consensus that the treatment of non-small cell lung cancer (NSCLC) prefers a combination of multidisciplinary comprehensive treatment and individualized treatment, which can significantly improve the prognosis of patients. Here, we report a female patient with recurrence-prone NSCLC. She had a decade-long disease course, during which the lesion recurred twice and finally cured with Multi-Disciplinary Treatment (MDT). An elderly female patient was admitted to the hospital after diagnosis of lung cancer, and treated with surgery and postoperative adjuvant chemotherapy. Five years later, suspicious lesions were found by computed tomography (CT) reexamination, and then confirmed tumor recurrence by puncture biopsy. Based on the genetic test results, gefitinib was used for subsequent targeted therapy, and the lesion gradually shrunk to disappear. However, the lesion appeared again two years later, after consultation the microwave ablation was adopted and the curative effect was excellent. At last, regular reexamination showed no abnormality, the patient has survived so far. The case proves the great benefit of multidisciplinary comprehensive treatment, especially microwave ablation for patient with recurrence-prone NSCLC. And the effect of systemic anti-tumor immune response induced by microwave ablation on lung cancer also needs to be further explored.

## Introduction

Based on reports, lung cancer has become the second common cancer in the world, and also the leading cause of cancer death (1). With the advancement of medical technology and in-depth research on lung cancer at the genetic level, various treatment modalities such as interventional therapy, targeted therapy and immunotherapy have been introduced (2). Nowadays, after evaluating the patient's body condition, histopathological type and molecular classification of the tumor, the scope of invasion and the trend of development, with a planned and rational application of surgery, radiotherapy, chemotherapy, interventional therapy, molecular targeted therapy and immunotherapy, it has become a consensus to adopt the mode of multidisciplinary comprehensive therapy combined with individualized therapy, which can maximize the survival time of patients. Here, we report a female patient with recurrence-prone NSCLC, whose disease course has lasted for almost ten years since first diagnosed. During this period, she experienced lesion recurrences twice and benefited from the multidisciplinary comprehensive treatment.

## Case presentation

**Figure 1:**
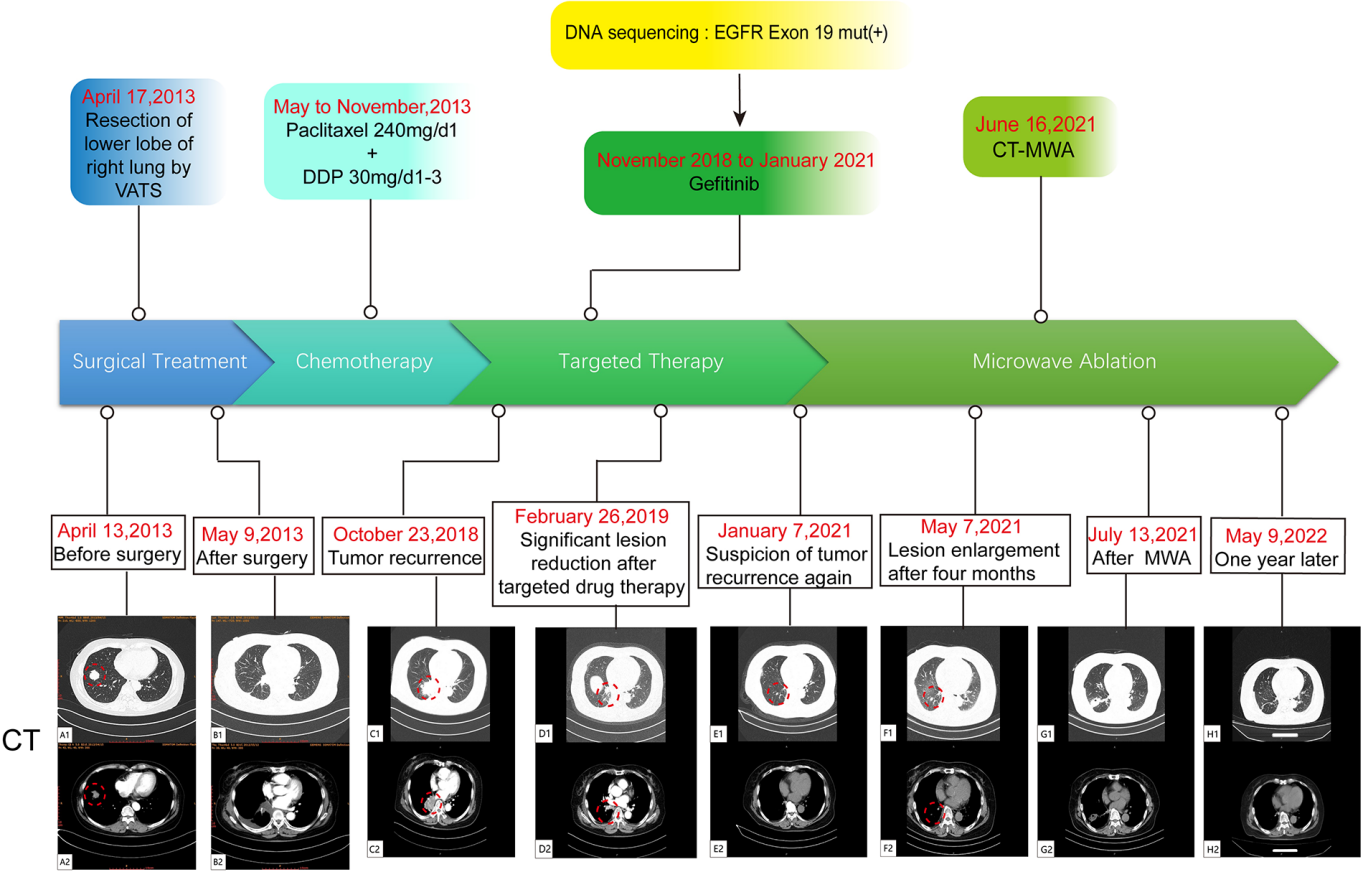
A flow chart of the entire treatment course. Red circles indicate the target lesion. VATS, video-assisted thoracic surgery; DDP, cis-diamminedichloroplatinum; CT-MWA, CT Guided Microwave Ablation.

**Figure 2:**
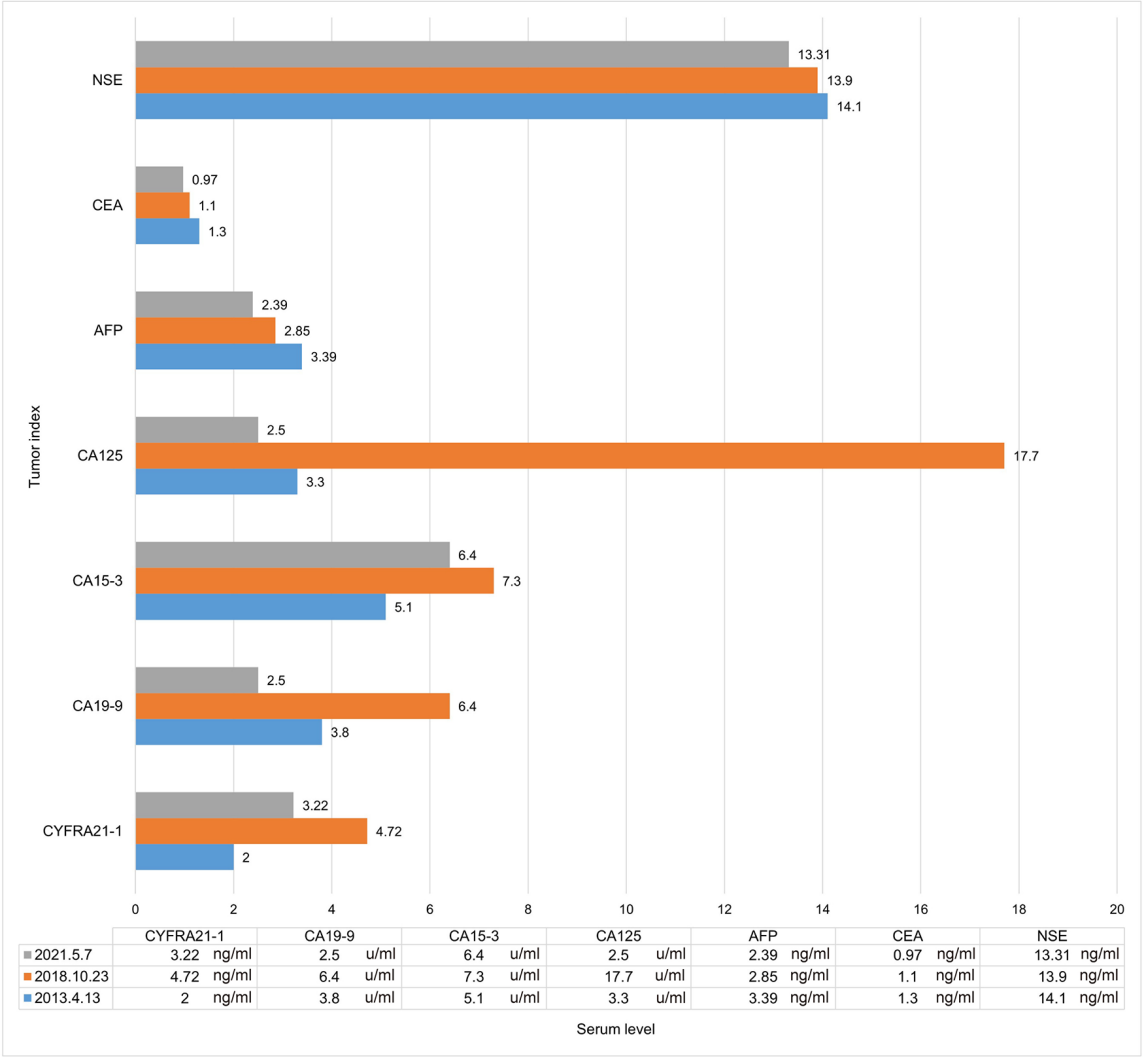
Tumor indicator levels when discovering lesions. AFP, alpha fetoprotein; CEA, carcinoembryonic antigen; NSE, neuron-specific enolase; CYFRA21-1, cytokeratin 19 fragment; CA125, carbohydrate antigen 125; CA19-9, carbohydrate antigen 19-9; CA15-3, carbohydrate antigen 15-3.

**Figure 3:**
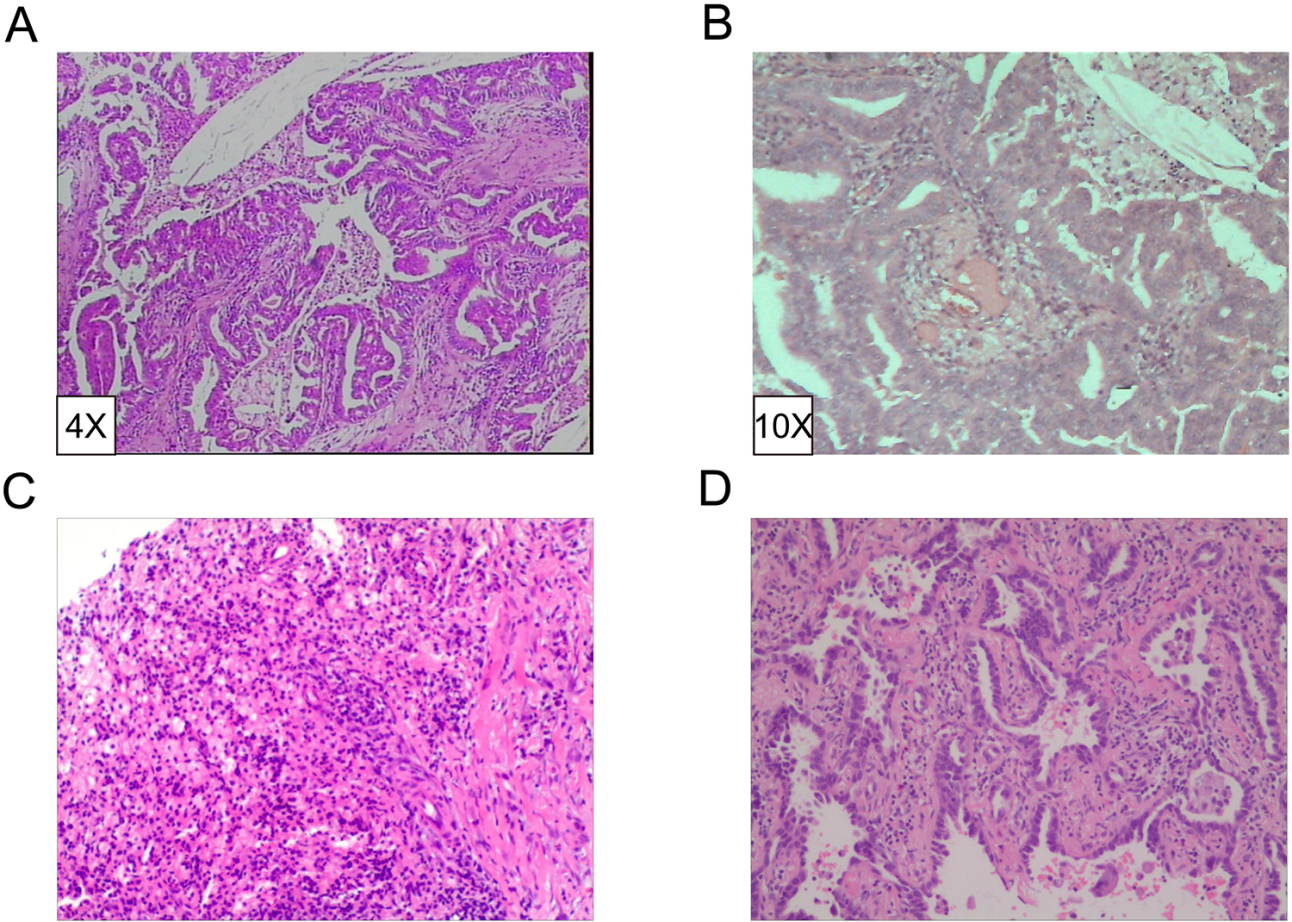
Pathological diagnosis of lesions. (**A,B**) Invasive adenocarcinoma grade II-III (alveolar dominant type). IHC revealed CK7+, CK5/6-, P63+, CK51+, TTF1+, SPA+, P16+, ER+ (20%), PR+ (10%), DOG1-. (HE4X/10X). (**C**) Chronic granulomatous inflammation with mechanization. (**D**) Invasive non-mucinous adenocarcinoma.

The patient, a 61-year-old female, was found to have a right lower lung occupancy of 2.0 cm in size by chest computed tomography (CT) on April 13, 2013 (Figure 1A), and then hospitalized. Tumor indicators: Alpha-fetoprotein (AFP): 3.39 ng/ml; Carcinoembryonic antigen (CEA): 1.30 ng/ml; Carbohydrate antigen (CA) 125: 3.30 U/ml; CA 19-9: 3.80 U/ml; Cytokeratin 19 fragments (CYFRA21-1): 2.0 ng/ml; Neuron-specific enolase (NSE): 14.10 ng/ml; CA 15-3: 5.1 U/ml (Figure 2). The video-assisted lobectomy of lung was performed under general anesthesia on April 17, 2013. During the operation, a mass in the outer basal segment of the right lower lobe was found, which was about 3 cm in diameter, medium texture and with pleural shrinkage on the surface. The postoperative pathology showed that the infiltrative adenocarcinoma of the right lower lung was grade II-III (alveolar predominant type), invading the dirty pleura (Figures 3A,B). “Group 10 lymph nodes” (0/1) and “Group 11 lymph nodes” (0/2). Immunohistochemistry: CK7+, CK5/6-, P63+, CK51+, TTF1+, spa+, P16+, ER+ (20%), PR+ (10%), DOG1−. Specimen genetic test results: EGFR Exon19 Deletion mutant; KRAS Exon2, Exon3 wild type; high expression in ERCC1, high expression in TS, low expression in RRM1, low expression in TUBB3. Pathological stage: PT2aN0M0, stage IB. The patient recovered well after surgery, and received six cycles of adjuvant chemotherapy with “paclitaxel 240 mg/d1 + DDP 30 mg/d1-3” from May 11, 2013. No obvious abnormality was found in the follow-up 5 years after operation.

On October 23, 2018, a soft tissue density occupancy in surgery area with pleural invasion was found by chest CT (Figure 1C), and the possibility of lung cancer recurrence was considered. Tumor indices: AFP: 2.85 ng/ml; CEA: 1.10 ng/ml; CA 125: 17.70 U/ml; CA 19-9: 6.40 U/ml; CYFRA21-1: 4.72 ng/ml; NSE: 13.90 ng/ml; CA 15-3: 7.30 U/ml (Figure 2). On October 30, 2018, the percutaneous aspiration biopsy of lung (PABL) was performed, and the pathology manifested chronic granulomatous inflammation with mechanization (Figure 3C). On November 6, 2018, positron emission tomography (PET-CT) showed a mass-like hyperdense shadow in the lower lobe of the right lung with abnormal increased glucose metabolism, considering the high possibility of lung cancer recurrence. On November 15, 2018, the second PABL was performed and the pathology of the right lower lung puncture tissue showed invasive non-mucinous adenocarcinoma (Figure 3D). In conjunction with the second molecular testing report: EGFR Exon19 Deletion mutant, the patient received gefitinib (Iressa) as the first-line treatment, and the right lung lesion was found rapidly shrunk on February 26, 2019 (Figure 1D), which proved that the treatment was effective. The subsequent two-year follow-up showed progressive disappearance of lesion.

On January 7, 2021, a lesion was found in the middle lobe of right lung during reexamination, about 8 mm (Figure 1E). On May 7, 2021, the lesion was found to be progressively enlarged, approximately 10 mm in size (Figure 1F). Radiographic examination suggested a high probability of tumor (Supplementary Figure S1). Tumor indicators: AFP: 2.39 ng/ml; CEA: 0.97 ng/ml; CA 125: 2.50 U/ml; CA 19-9: 2.50 U/ml; CYFRA21-1: 3.22 ng/ml; NSE: 13.31 ng/ml; CA 15-3: 6.40 U/ml (Figure 2). Whole-body examination was applied to exclude the presence of metastasis, including head-thorax-abdomen CT, bone scan and so on. Considering the higher risk of surgical bleeding and the relatively less benefit of reoperation after recurrence, CT Guided Microwave Ablation was performed on June 16, 2021, and the pathological biopsy during microwave therapy was confirmed to be adenocarcinoma (Supplementary Figure S2). On July 13, 2021, the chest CT showed that the right middle lung mass with peripheral speckles and cords, which was considered as changes after microwave ablation (Figure 1G). With regular follow-up to May 2022 (Figure 1H), no obvious abnormality was found, which proves a good outcome. The whole therapy process of the patient is shown in Figure 1.

## Discussion

From the last century to present, surgery has always been the best option for the treatment of lung cancer. According to the principles of surgery, lobectomy is the best option for NSCLC patients with good tolerance, while hilar and mediastinal lymph node dissection should also be a routine part of the procedure (3). Several reports have indicated that postoperative adjuvant chemotherapy based on cisplatin can result in a better prognosis (4). The patient's condition also remained stable after six cycles of adjuvant chemotherapy. Although early stage of NSCLC can be resected radically, the postoperative recurrence rate can be 30% to 55%. According to previous research, some concealed micrometastatic cancer cells already exist systematically during surgery, and standard staging methods cannot encompass these cancer cells. Secondly, the spread of tumor cell may also occur during surgical management of the tumor (5). Prior studies have also proved that compared with upper lobectomy, the non-recurrence rate (excluding non-specific death) and overall survival after lower lobectomy are significantly worse (6).

Nevertheless, owing to the tumor genetic test, the patient has a mutation in EGFR Exon19, which meets the criteria of targeted therapy. EGFR, as one kind of tyrosine kinase receptor, its mutations and elevated levels of expression are associated with a variety of cancers, particularly in lung cancer (7). Research have shown that about 15% western patients and 35% Asian patients have EGFR mutations, the most common of which is exon 19 deletion (Del19) (8). A significant increase was recorded in the effectiveness of the therapy in NSCLC patient after applying gefitinib (first-generation tyrosine kinase inhibitor), and the lesion decreased rapidly. Nevertheless, almost all NSCLC patients with EGFR mutations will eventually develop drug resistance to TKI therapy (9). The underlying mechanisms include: 1, EGFR-dependent on-target resistance; 2, bypass-activated off-target resistance; 3, histological type transformation (10). Clinical data also showed that the median progression-free survival after gefitinib treatment was about 10 months (11).

**Figure 4:**
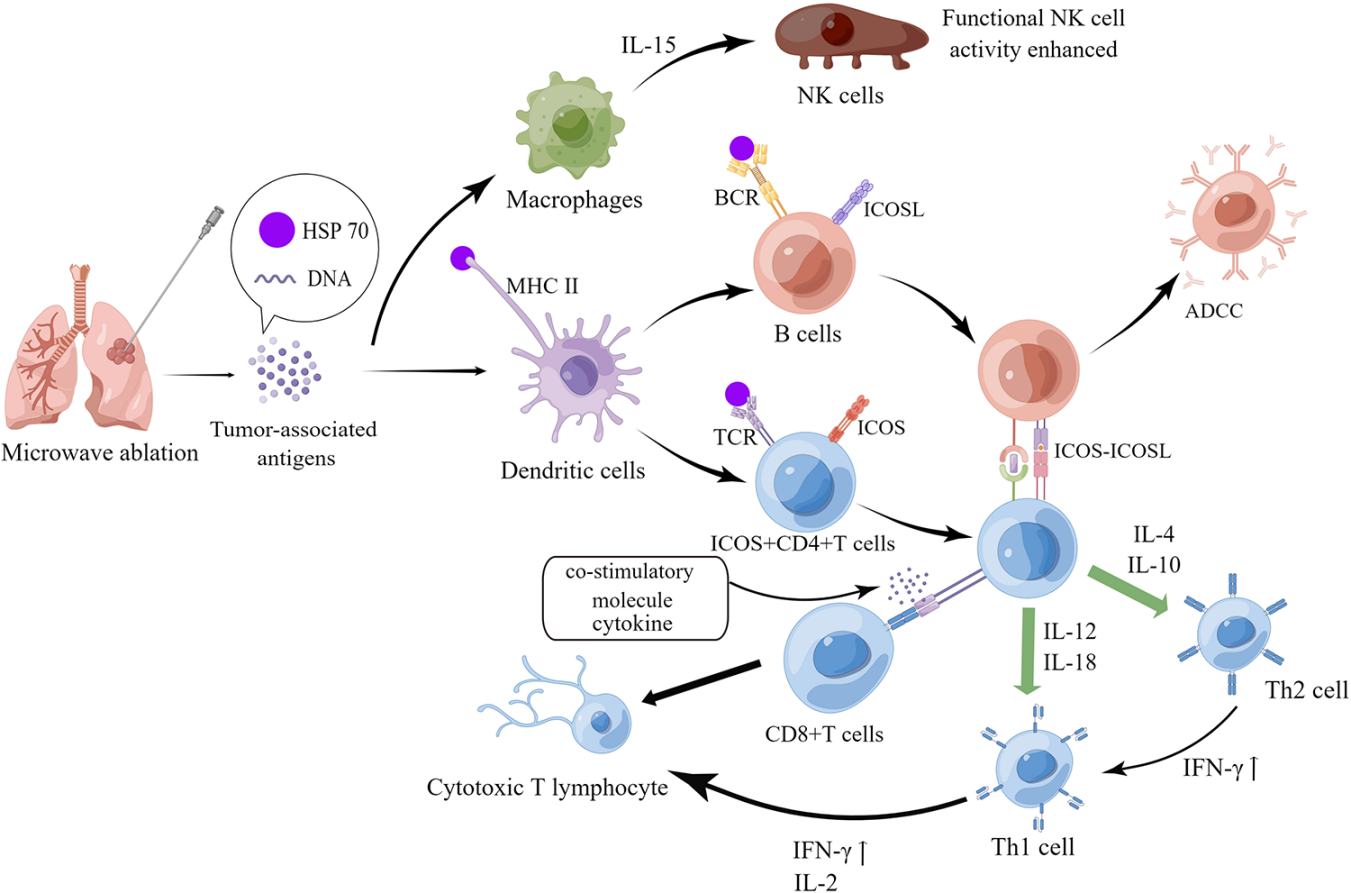
Mechanism of systemic anti-tumor immune response induced by microwave ablation. The figure was drawn by Figdraw.

After the second recurrence of lesion, the patient opted for minimally invasive interventional therapy. Percutaneous image-guided thermal ablation (IGTA), including radiofrequency ablation (RFA), microwave ablation (MWA), and cryoablation, is a minimally invasive treatment for NSCLC and thoracic metastatic tumors (12). Several researches have stated that compared with drug therapy, carefully selected local ablation for lung cancer can also improve overall survival and progression-free survival (13). A number of recent studies have noted that in addition to thermal injury, local thermal ablation can induce abscopal effect—one kind of systemic anti-tumor immune response, which may be another mechanism of tumor cell destruction and death. According to previous studies, in MWA-induced immune response, B cells were significantly activated, whose antigen presentation capacity was significantly enhanced. And then tumor antigens were presented to CD4 + T cells, which activated CD4 + T cells and produced more IFN-*γ* through inducible costimulatory molecule (ICOS-ICOSL) pathway, driving the balance of Th1/Th2 to Th1, and further aiding CD8 + T cell activation to generate CTL, ultimately leading to an enhanced anti-tumor capacity of the systemic immune system (14–17). In addition, macrophages are activated by tumor-associated antigens and produce IL-15, which activates NK cells, generally enhance functional activity of NK cells, and then facilitates the destruction of tumor cells (18). The specific immune mechanisms are shown in Figure 4. The patient then continued maintenance treatment with targeted drugs and is now in stable condition. If the patient relapses in future, we will perform puncture biopsy again for pathological typing and genetic testing (tissue and blood) to determine whether drug-resistant genes are developed, and then consider other treatment options such as stereotactic body radiation therapy (SBRT).

The entire course of this patient's disease lasted 9 years and 4 months, during which two relapses occurred and treated with different methods. The whole process was rather complicated, while the final prognosis was well.

In conclusion, multidisciplinary comprehensive diagnosis and treatment of recurrence-prone lung cancer has tremendous value in improving the survival of lung cancer patients by developing precise, personalized and appropriate treatment plans. And the effect of systemic anti-tumor immune response induced by microwave ablation on lung cancer also needs to be further explored.

## Data Availability

The original contributions presented in the study are included in the article/**Supplementary Material**, further inquiries can be directed to the corresponding author/s.
