# Supplementary material for: Recurrent lung adenocarcinoma benefits from microwave ablation following multidisciplinary treatments: A case with long-term survival
Source: Front Surg. 2023 Jan 6;9:1038219. doi: 10.3389/fsurg.2022.1038219 (PMC9852634; doi:10.3389/fsurg.2022.1038219)
Supplement: Supplementary file 1 [file Datasheet1.pdf]

CARE case report guidelines

CARE Checklist of information to include when writing a case report

(cc) BY-NC-ND

Topic	Item No	Checklist item description	Reported on Page Number/Line Number	Reported on Section/Paragraph
Title	1	The diagnosis or intervention of primary focus followed by the words “case report”	Page1/Line1-2	Title/1
Key Words	2	2 to 5 key words that identify diagnoses or interventions in this case report, including "case report"	Page1/Line22	Keywords/1
Abstract (Structured summary)	3a	Background: state what is known and unknown; why the case report is unique and what it adds to existing literature.	Page2/Line24-32	Abstract/1
	3b	Case Description: describe the patient’s demographic details, main symptoms, history, important clinical findings, the main diagnosis, interventions, outcomes and follow-ups.	Page2/Line24-42	Abstract/1
	3c	Conclusions: summarize the main take-away lesson, clinical impact and potential implications.	Page2/Line24-42	Abstract/1
Introduction	4	One or two paragraphs summarizing why this case is unique **(may include references)**	Page3/Line44-58	Introduction/1
Patient Information	5a	De-identified patient specific information	Page3/Line60-80	Case presentation/1
	5b	Primary concerns and symptoms of the patient	Page3/Line60-80	Case presentation/1
	5c	Medical, family, and psycho-social history including relevant genetic information	Page3/Line60-80	Case presentation/1
	5d	Relevant past interventions with outcomes	Page3/Line60-80	Case presentation/1
Clinical Findings	6	Describe significant physical examination (PE) and important clinical findings	Page3/Line60-80	Case presentation/1
Timeline	7	Historical and current information from this episode of care organized as a timeline	N/A	N/A
Diagnostic Assessment	8a	Diagnostic testing (such as PE, laboratory testing, imaging, surveys).	Page3/Line60-80	Casepresentation/1
	8b	Diagnostic challenges (such as access to testing, financial, or cultural)	Page3-4/Line81-96	Case presentation/2
	8c	Diagnosis (including other diagnoses considered)	Page3-4/Line81-96	Case presentation/2
	8d	Prognosis (such as staging in oncology) where applicable	Page3-4/Line81-96	Case presentation/2
Therapeutic Intervention	9a	Types of therapeutic intervention (such as pharmacologic, surgical, preventive, self-care)	Page4/Line97-108	Case presentation/3
	9b	Administration of therapeutic intervention (such as dosage, strength, duration)	Page4/Line97-108	Case presentation/3
	9c	Changes in therapeutic intervention (with rationale)	Page4/Line97-108	Case presentation/3

Follow-up and Outcomes	10a	Clinician and patient-assessed outcomes (if available)	**Page4/Line97-108**	**Case presentation/3**
	10b	Important follow-up diagnostic and other test results	**Page4/Line97-108**	**Case presentation/3**
	10c	Intervention adherence and tolerability (How was this assessed?)	**Page4/Line97-108**	**Case presentation/3**
	10d	Adverse and unanticipated events	**Page4/Line97-108**	**Case presentation/3**
Discussion	11a	A scientific discussion of the strengths AND limitations associated with this case report	**Page4/Line111-124**	**Discussion/1**
	11b	Discussion of the relevant medical literature **with references**	**Page4/Line125-137**	**Discussion/2**
	11c	The scientific rationale for any conclusions (including assessment of possible causes)	**Page4-5/Line138-158**	**Discussion/3**
	11d	The primary "take-away" lessons of this case report (without references) in a one paragraph conclusion	**Page5/Line159-166**	**Discussion/4**
Patient Perspective	12	The patient should share their perspective in one to two paragraphs on the treatment(s) they received	**N/A**	**N/A**
Informed Consent	13	Did the patient give informed consent? Please provide if requested	**Yes** ☑	**No** ☐

Please leave this space alone as it will be supplemented by the editorial office when needed.
