# Supplementary material for: Recurrent lung adenocarcinoma benefits from microwave ablation following multidisciplinary treatments: A case with long-term survival
Source: Front Surg. 2023 Jan 6;9:1038219. doi: 10.3389/fsurg.2022.1038219 (PMC9852634; doi:10.3389/fsurg.2022.1038219)
Supplement: Supplementary file 2 [file Datasheet2.pdf]

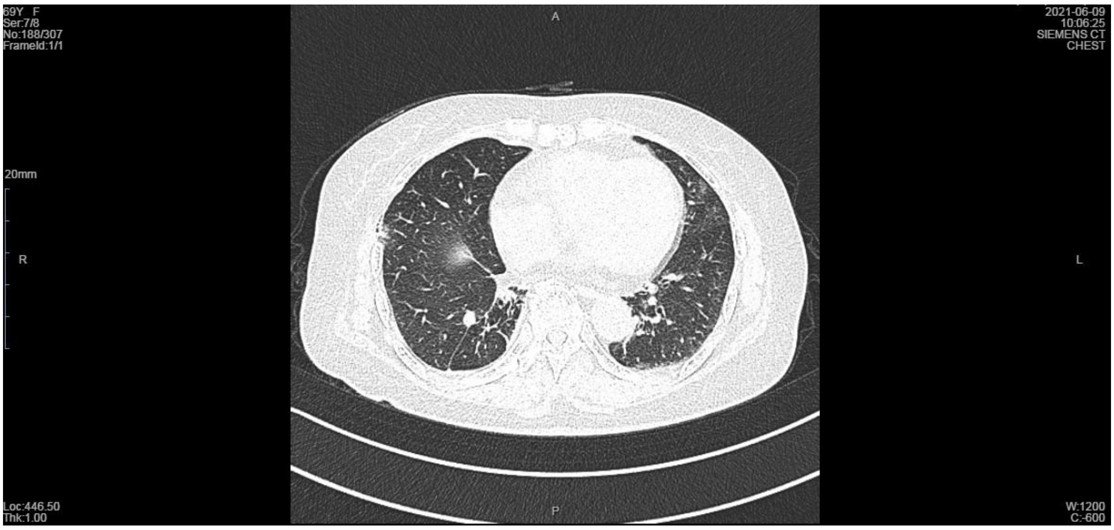

Supplementary Figure 1. The CT image of chest before microwave ablation in June 2021.

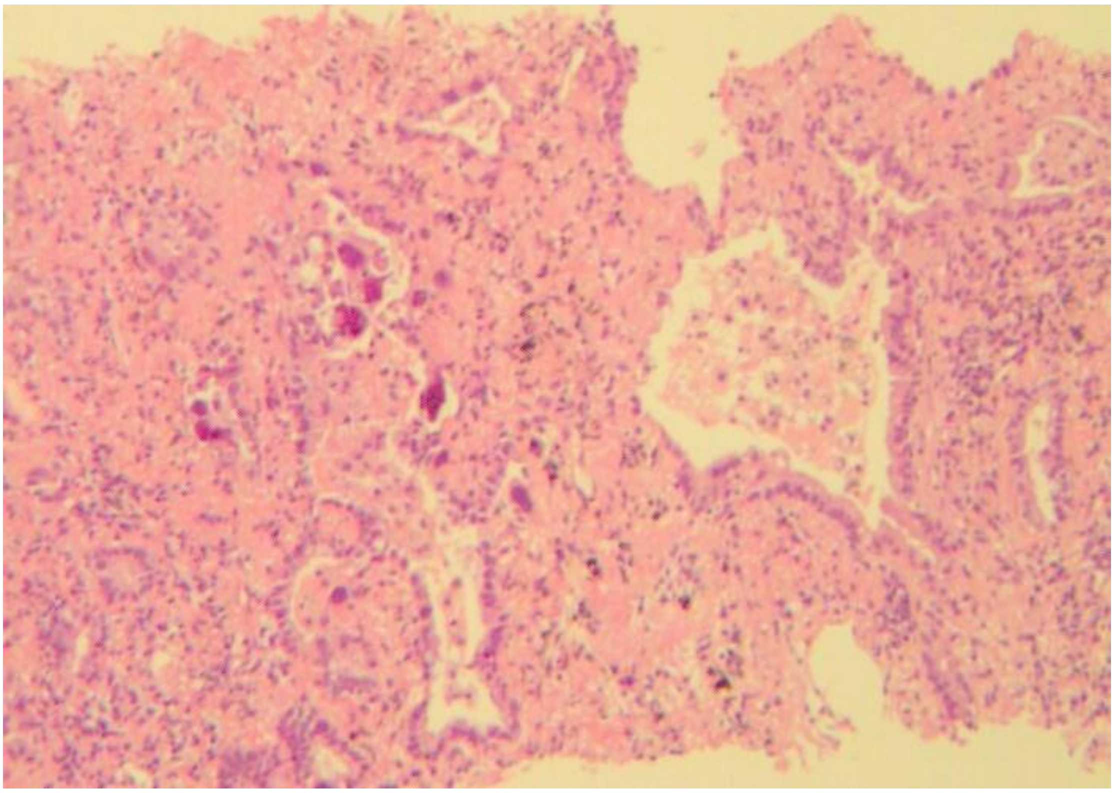

Supplementary Figure 2. The result of pathological biopsy during microwave therapy showed adenocarcinoma.
